# Deep Neural Network Sleep Scoring Using Combined Motion and Heart Rate Variability Data

**DOI:** 10.3390/s21010025

**Published:** 2020-12-23

**Authors:** Shahab Haghayegh, Sepideh Khoshnevis, Michael H. Smolensky, Kenneth R. Diller, Richard J. Castriotta

**Affiliations:** 1 Department of Biostatics, T.H. Chan School of Public Health, Harvard University, Boston, MA 02115, USA; 2 Department of Biomedical Engineering, Cockrell School of Engineering, The University of Texas at Austin, Austin, TX 78712, USA; sepideh@utexas.edu (S.K.); Michael.H.Smolensky@uth.tmc.edu (M.H.S.); kdiller@mail.utexas.edu (K.R.D.); 3 Department of Internal Medicine, Division of Cardiology McGovern School of Medicine, The University of Texas Health Science Center at Houston, Houston, TX 77030, USA; 4 Division of Pulmonary, Critical Care and Sleep Medicine, Keck School of Medicine, University of Southern California, Los Angeles, CA 90033, USA; Richard.Castriotta@med.usc.edu

**Keywords:** wrist actigraphy, sleep, artificial intelligence, deep learning, Convolutional Neural Network (CNN), time series classification, Long-Short-Term Memory (LSTM)

## Abstract

*Background:* Performance of wrist actigraphy in assessing sleep not only depends on the sensor technology of the actigraph hardware but also on the attributes of the interpretative algorithm (IA). The objective of our research was to improve assessment of sleep quality, relative to existing IAs, through development of a novel IA using deep learning methods, utilizing as input activity count and heart rate variability (HRV) metrics of different window length (number of epochs of data). *Methods:* Simultaneously recorded polysomnography (PSG) and wrist actigraphy data of 222 participants were utilized. Classic deep learning models were applied to: (a) activity count alone (without HRV), (b) activity count + HRV (30-s window), (c) activity count + HRV (3-min window), and (d) activity count + HRV (5-min window) to ascertain the best set of inputs. A novel deep learning model (Haghayegh Algorithm, HA), founded on best set of inputs, was developed, and its sleep scoring performance was then compared with the most popular University of California San Diego (UCSD) and Actiwatch proprietary IAs. *Results:* Activity count combined with HRV metrics calculated per 5-min window produced highest agreement with PSG. HA showed 84.5% accuracy (5.3–6.2% higher than comparator IAs), 89.5% sensitivity (6.2% higher than UCSD IA and 6% lower than Actiwatch proprietary IA), 70.0% specificity (8.2–34.3% higher than comparator IAs), and 58.7% Kappa agreement (16–23% higher than comparator IAs) in detecting sleep epochs. HA did not differ significantly from PSG in deriving sleep parameters—sleep efficiency, total sleep time, sleep onset latency, and wake after sleep onset; moreover, bias and mean absolute error of the HA model in estimating them was less than the comparator IAs. HA showed, respectively, 40.9% and 54.0% Kappa agreement with PSG in detecting rapid and non-rapid eye movement (REM and NREM) epochs. *Conclusions:* The HA model simultaneously incorporating activity count and HRV metrics calculated per 5-min window demonstrates significantly better sleep scoring performance than existing popular IAs.

## 1. Introduction

Polysomnography (PSG) is considered the gold standard for sleep evaluation. However, this method is not always an option. PSG requires bulky instrumentation and wiring of the patient making the sleep environment unnatural. Moreover, novelty of the sleep laboratory environment and night-to-night difference in sleep quality might confound accurate sleep assessment [1,2]. Furthermore, differences in the scoring of PSG data between and within raters are potential sources of error [3]. Finally, PSG studies are expensive, thereby resulting in the exclusion of some patients and discouraging longitudinal assessment. Questionnaires and diaries are inexpensive and easy to use methods for large-scale studies. Sleep questionnaires, however, depend on the retrospective recall of patients and this can introduce biases [4]. Sleep diaries lack accuracy because of poor awareness of patients of certain events, such as the exact time of falling asleep and number of awakenings during sleep [5]. More importantly, these methods are incapable of ascertaining reliable information on any of the main sleep parameters, such as time spent awake after sleep onset (WASO). Additionally, non-invasive motion sensors, such as passive InfraRed motion sensor [6] or bed sensors, have been used for the purpose of sleep assessment; however, these methods have not yet been studied extensively. Wrist actigraphy, due to its low cost and ability to estimate the main sleep parameters in an unobtrusive manner, is a popular method for assessing the sleep of persons residing in free-living conditions. Nevertheless, the accuracy of this method depends on both the quality of the hardware (sensor) technology and the performance of interpretative algorithms (IA) in scoring of sleep by sensed movement.

Wrist actigraphy is considered by The American Academy of Sleep Medicine as a method with acceptable accuracy to evaluate adult sleep patterns [7], although without specific recommendation of specific scoring IA. Even though actigraphy has high sensitivity in detecting sleep (between 0.87 to 0.99), its specificity is low (between 0.28 to 0.67) [8], depending upon its hardware and IA. Sadeh [9], Cole-Kripke [10], rescored Cole-Kripke [10,11], and University of California San Diego (UCSD) [12] IAs are the ones most commonly used to process actigraphy data and score wake/sleep epochs. To utilize these IAs, as a first step, raw accelerometer data need to be pre-processed and converted into activity counts. The method by which sensed raw accelerometer data are processed is called “mode of operation”. Proportional Integrating Measure (PIM), Time Above Threshold (TAT), and Zero-Crossing Mode (ZCM) are the most common operational modes of actigraphy. ZCM is the number of voltage crosses in response to body movements that exceed threshold per given time interval (epoch), whereas TAT is the duration of time voltage exceeds a chosen threshold value in response to movements per given epoch. Lastly, PIM is the total area under the time-movement curve during any given epoch, regardless of voltage sign [13]. The Cole-Kripke [10], rescored Cole-Kripke [10,11], and Sadeh [9] IAs utilize the ZCM to score a specific epoch as wake or sleep. The UCSD algorithm is the only well-known IA that is equally applicable to data derived by the PIM, TAT, or ZCM modes [12]. It progressively rates each successive epoch as wake or sleep on the basis of a 7-min data span—the current 1 min, the 4 preceding minutes, and the 2 subsequent minutes [12]. Recently, researchers have increasingly focused on improving the performance of the wrist actigraphy method by incorporating more input features, in addition to activity count indices, like heart rate (HR) or heart rate variability (HRV). Studies have shown an association between sleep stages and HR and HRV [14]; both decrease during sleep stages 1 to 4 and increase during rapid eye movement (REM) sleep [14,15]. There is also significant increase in HRV low frequency/high frequency ratio (LF/HF) during REM sleep [14,16].

We previously compared the performance of the four commonly used conventional wrist actigraphy IAs [17] and also more advanced IAs [18], such as those used by the Fitbit fitness trackers [19,20] in assessing sleep. We also studied the effect of the selected mode of operation on sleep scoring performance [21]. The purpose of our most recent work summarized in this article is to: (1) report the effect of utilizing HRV measures plus actigraphy count data in combination on sleep scoring performance in contrast to utilizing actigraphy count data only, (2) compare the effect of incorporating different HRV period lengths on sleep scoring performance, (3) introduce the Haghayegh algorithm (HA) that simultaneously incorporates HRV measures and actigraphy data into a deep learning model to score epochs as wake and sleep, and (4) evaluate the performance of the novel HA relative to the Actiwatch Spectrum proprietary IA and also the UCSD IA in accord with standards and terminologies established by national sleep and technology organizations for wearable wristband devices [22].

## 2. Methods

### 2.1. Dataset

We used the PSG and wrist actigraphy data of the Multi-Ethnic Study of Atherosclerosis (MESA) sleep [23,24] studies that entailed the simultaneous collection of wrist movement sensed by the Actiwatch Spectrum (Philips Respironics, Murrysville, PA, USA) and PSG recordings obtained by a commercial in-home PSG system (Compumedics Somte System, Compumedics Ltd., Abbotsford, Australia). Data of the synchronized PSG studies of various rated quality and actigraphy (PIM mode only) of the MESA are available for 1835 participants through The National Sleep Research Resource website (sleepdata.org). In order to have an accurate reference to compare the performance of sleep scoring models, we only used PSG data whose overall quality of data were rated by certified PSG scorers as outstanding, i.e., the signal of all of the channels being rated good for the entire duration of sleep that lasted longer than 6 h (N = 253).

### 2.2. Heart Rate Variability Features

The electrocardiography (ECG) signal of the PSG was used to extract HRV metrics. The ECG was recorded with sampling rate of 256 HZ using Ag/AgCl patch electrodes. Since the purpose of this study was to compare the effect of incorporating different HRV period durations on sleep scoring performance and also developing a novel IA based on the combination of activity count and HRV, we decided to use the most accurate signal for calculating HRV, i.e., the ECG signal, even though the same HRV metrics can be also derived by plethysmography. In total, 17, i.e., 9 time domain and 8 frequency domain, HRV metrics were extracted per 30-s epoch using Kubios HRV Premium (ver 3.3, Kubios, Kuopio, Finland) by applying automatic QRS detection and artifact correction algorithms [25]. Only the data of sleep studies with a percentage of artifact <5% (N = 222) of the entire recording period were utilized. Table 1 presents the list of the HRV metrics considered, along with their units and definitions [26,27].

HRV metrics are significantly affected by the window duration selected to derive them, i.e., number of data epochs comprising the HRV calculation. In the literature, HRV recording period length (window) shorter than 5 min is considered as ultra-short-term measurement and that of ~5-min as short-term measurement [26]. In this study, we compared 3 different window lengths of HRV calculation: (1) 30-s window, which is equal to the epoch size of the PSG sleep scoring estimation, (2) 5-min window, considered as short-term measurement, and one between these two, i.e., 3-min window.

### 2.3. Participants

The sleep data of 222 participants met the inclusion and exclusion criteria for analysis. Table 2 summarizes the characteristics of the subjects of the sleep study population.

### 2.4. Deep Learning Algorithm Training and Selection

**Figure 1:**
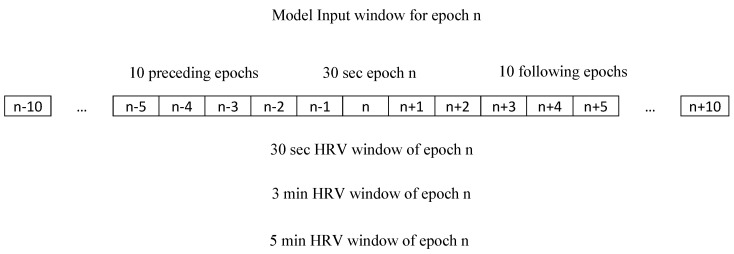
Illustrative example of different heart rate variability (HRV) window lengths used to calculate the HRV metrics of epoch n. The HRV metrics plus wrist activity count of all the epochs from epoch n − 10 to epoch n + 10 are utilized as model input window to score epoch n. Scoring of successive epochs of the respective time series is done in progressive steps in which 30 s of new data are added to the frontside of the window as 30 s of old data are deleted from the backside of the window.

The database of the 222 subjects was randomly divided into testing (N = 77 subjects; 35%) and training/validation (N = 145 subjects; 65%) sets. None of the test set data was utilized for training, tuning, or selection of the analytical models. The raw time series data of activity count and HRV metrics mode channels as input into the model centered around each successive 30-s epoch of a 10.5-min time window, i.e., 10 epochs preceding and 10 epochs following the centered 30-s (see Figure 1). The effect of combining the HRV and activity data plus effect of HRV window length, i.e., 30-s vs. 3-min vs. 5-min, was explored using four classic deep learning architectures. In this manner, residual network [29,30], fully convolutional neural networks [29,30], encoder [29,31], and time Le-Net [29,32] were applied to datasets of activity count only, activity count + 30-s HRV, activity count + 3-min HRV, and activity count + 5-min HRV. The models were trained and evaluated on a training dataset using a 5-fold cross-validation process. A random search over the hyperparameters, using the Mcfly software package [33], was performed to generate 50 sets of convolutional neural network (CNN) [34] models and 50 sets of deep learning convolutional, long-short-term, memory (DeepConvLSTM) [35] models. The randomly selected hyperparameters were regularization rate, learning rate, number of convolution layers, number of filters per layer, number of hidden nodes, and number of long-short-term memory (LSTM) layers (only for DeepConvLSTM) [33]. Some 70% of the training/validation dataset was randomly used to train the total of 100 models, and the remaining 30% was used to evaluate the models. The top 5 models of best performance, i.e., lowest validation loss values, were further evaluated with a 5-fold cross-validation strategy using the training/validation dataset, exclusive of the test dataset. The deep learning IAs were produced using the TensorFlow [36,37] and Keras library [38] in Python (version 3.7).

### 2.5. Statistical Analyses

Since the start and end time of each PSG was unspecified per subject study, the pulse oximetry (SpO2) data of the PSG study were utilized as a surrogate biomarker of both the start time, i.e., the first 5-min block that contained ≥30-s of SpO2 values, and end time, i.e., the last 5-min block that contained ≥30-s of SpO2 values. Accuracy, specificity, sensitivity, and Cohen’s Kappa in detecting sleep epochs were calculated by equations provided in Appendix A for each individual subject for the Actiwatch proprietary, UCSD, and proposed deep learning IAs. These metrics were additionally calculated in detecting REM and Non-Rapid Eye Movement (NREM) sleep epochs for the proposed deep learning IA only, since the other two IAs lack the capability of estimating sleep stages. Bland–Altman plots [39] were generated to compare performance between the PSG and each IA in estimating the individual sleep quality parameter (See Table 3 for definition of each one). A negative value of bias indicates overestimation of the PSG value by the IA. Linear regression analysis was applied to test the null hypothesis of absence of significant trend in Bland–Altman plots, i.e., no trend in difference between values determined by PSG and given IA across subjects (y-axis of Bland–Altman plot) relative to the overall mean of the PSG and given IA values (x-axis of Bland–Altman plot) [40]. The null hypothesis that the estimated value of a sleep parameter by each IA did not significantly differ from the reference PSG value was paired t-tested. Additionally, the null hypothesis that the amount of bias in an estimated sleep parameter by the proposed deep learning method did not vary significantly from that estimated by each of the other two IAs was assessed by t-test. We also calculated the Mean Absolute Error (MAE) as the average of the absolute values of difference between PSG and IAs per sleep parameter. Python (version 3.7) and MATLAB (version R2020a) were used for data analyses. P values less than 0.05 was considered as evidence of a statistically significant difference.

## 3. Results

### 3.1. Comparison of Different HRV Period (Window) Lengths

Table 4 reports the comparison with PSG of the classic deep learning architectures applied to activity count only, activity count + 30-s HRV, activity count + 3-min HRV, and activity count + 5-min HRV in scoring epochs as sleep or wake. As apparent from Table 4, the combination of activity count plus 5-min HRV provided highest accuracy and Kappa agreement.

### 3.2. Best Deep Learning Model’s Architecture

A DeepConvLSTM, consisting of 9 convolutional layers, each followed by a batch normalization and 4 LSTM layers after the last convolutional layer, proved to be the best model. Binary cross entropy was applied as a loss function for the classification task. The kernel regularization method was used to prevent overfitting, and the minimum validation loss was used to identify the model weights. The Supplementary Materials Table S1 provides the configuration of the best model.

### 3.3. Overall Estimate of Sleep Parameters

Table 3 lists the overall mean and standard deviation of the sleep parameters assessed by PSG as well as by Actiwatch with data analyzed, respectively, by its proprietary IA, UCSD IA, and proposed HA. Mean sleep onset latency (SOL) varied from ~10 min by the Actiwatch proprietary IA to ~54 min by the proposed deep learning HA, while the reference PSG was ~61 min. WASO varied from ~59 min by the Actiwatch proprietary IA to ~116 min by the UCSD IA, while the reference PSG was ~83-min. Total sleep time (TST) ranged from ~359 min by the UCSD IA to ~442 min by the Actiwatch proprietary IA, while the reference PSG was ~368 min. Finally, sleep efficiency (SE) varied from ~71% by the UCSD IA to ~87% by the Actiwatch proprietary IA, while the reference PSG was ~73%. Overall, estimation of the sleep parameters by the HA deep learning method displayed greatest similarity to PSG; in contrast, average values derived by the Actiwatch proprietary IA were most divergent from PSG values.

### 3.4. Epoch-by-Epoch Comparisons

Table 5 presents the accuracy, sensitivity, specificity, and kappa agreement of each IA in detecting sleep (vs. wake) epochs, in reference to PSG. Additionally, these metrics are reported for the HA deep learning algorithm in detecting REM (vs. NREM sleep + wake) epochs and also NREM sleep (vs. REM sleep + wake) epochs in reference to PSG. The deep learning HA showed highest accuracy (84.5%), specificity (70.0%), and Cohen’s Kappa (58.7%), while the Actiwatch proprietary IA showed highest sensitivity (95.5%) in detecting sleep epochs.

### 3.5. Performance in Estimating Sleep Parameters

**Figure 2:**
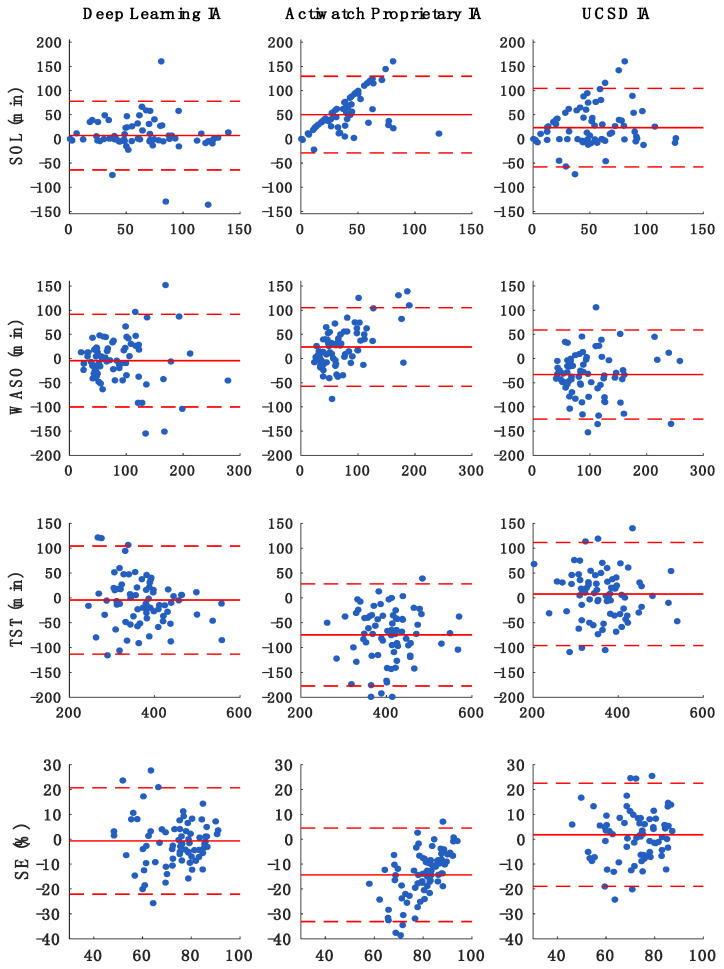
Bland–Altman plots of sleep parameters assessed by polysomnography (PSG) vs. Actiwatch proprietary, UCSD, and deep learning Haghayegh algorithms; x axis displays mean value of each individual sleep parameter (average of PSG and IA derived values) and y axis displays between-device/method difference (PSG minus IA). Positive bias indicates underestimation of PSG-derived values by the IA. Solid lines denote bias and dashed lines upper and lower limits of agreement (bias ± 1.96*SD). Abbreviation: IA: interpretative algorithm; SE: sleep efficiency; SOL: sleep onset latency; TST: total sleep time; WASO: wake after sleep onset.

Figure 2 presents the Bland–Altman plots that compare PSG, as reference, with the proposed deep leaning HA, Actiwatch proprietary IA, and UCSD IA for the sleep parameters of SOL, WASO, TST, and SE, and Table 6 summarizes the details of these comparisons. In reference to PSG, there was no statistically significant bias in the estimation of any of the sleep parameters—SOL, WASO, TST, and SE—by the HA. In contrast, SOL was underestimated by the Actiwatch proprietary IA (~50 min) and UCSD (~23-min); WASO was underestimated by the Actiwatch proprietary IA (~24 min) and overestimated by UCSD (~33-min); TST was overestimated by the Actiwatch proprietary IA (~75-min) and underestimated by UCSD (~8 min); and SE was overestimated by the Actiwatch proprietary IA (~14%) and underestimated by UCSD (~2%). In comparison to the PSG reference, the amount of bias in the estimated SOL, WASO, TST, and SE parameters by the deep learning HA was significantly smaller than that of the UCSD and Actiwatch proprietary IAs. Moreover, the MAE of each of the sleep parameters was smallest by the HA relative to the UCSD and Actiwatch proprietary IAs. Regression analysis of Bland–Altman data revealed significant positive slope in bias for the sleep parameters of SOL, WASO, and SE derived from the Actiwatch proprietary IA. The amount of bias in SOL and WASO estimated by Actiwatch proprietary IA increased as mean values increased, while that in SE decreased as mean values increased. There was a significant negative slope in bias for the TST derived from the HA, meaning that the amount of bias in estimating TST was smallest for the middle range values of TST (Supplementary Materials Table S2).

### 3.6. REM and NREM Sleep Detection/Scoring

The UCSD and Actiwatch proprietary IAs are incapable of estimating sleep stages, but the HA is. Table 5 presents epoch-by-epoch comparison of the proposed deep learning HA with PSG in detecting REM and NREM sleep epochs. The deep learning HA performs better in detecting NREM epochs (Kappa agreement = 54.0) than REM epochs (Kappa agreement = 40.9). Table 6 shows the performance of the deep learning HA in estimating the total duration of REM and NREM sleep. The deep learning HA significantly underestimated the REM sleep duration and overestimated Non-REM sleep duration.

## 4. Discussion

The purpose of this project was to: (1) investigate the effect of combining activity count and HRV metrics on sleep scoring performance, (2) compare the effect of different HRV period lengths (windows) on sleep scoring performance, (3) devise a novel IA to improve sleep scoring performance of wrist actigraphy based upon deep learning methods that simultaneously incorporates HRV and activity count values, and (4) compare performance of this novel IA with existing UCSD and Actiwatch proprietary IAs.

In regard to the first and second study objectives, we applied four classic deep learning architectures to only activity count data and, in addition, the combination of activity count with 30-s, 3-min, and 5-min windows of HRV metrics data to perform epoch-by-epoch comparisons between the most utilized IAs vs. PSG as reference in scoring sleep vs. wake. The IA comprised of the combined activity counts plus 5-min HRV window was termed the deep learning Haghayegh algorithm (HA) and provided the highest accuracy and agreement with the reference PSG in detecting sleep epochs. Both the time domain and frequency domain of the HRV metrics were significantly affected by the window duration, i.e., number of data epochs comprising the HRV calculation. Some HRV metrics have a recommended recording period length (window) of at least 5-min, because ultra-short period lengths fail to provide the same values as the 5-min period length [26].

Regarding the third and fourth study questions, we developed a deep learning model that incorporates both activity count and 5-min HRV metrics to score wrist actigraphy data. Per epoch comparison of the novel HA relative to the PSG in the ability to properly score sleep epochs disclosed accuracy of 84.5%, sensitivity of 89.5%, specificity of 70.0%, and Kappa agreement of 58.7%. We further compared the performance, relative to PSG, of the HA against the UCSD and also the Actiwatch proprietary IAs. Our proposed deep learning HA exhibited highest accuracy (respectively, 6.2% and 5.3% higher than the UCSD and Actiwatch proprietary IAs), specificity (respectively, 8.2% and 34.3% higher than the UCSD and Actiwatch proprietary IAs), and Kappa agreement (respectively, 16% and 23% higher than the UCSD and Actiwatch proprietary IA). The sensitivity of the proposed deep learning HA was higher than that of the UCSD IA (6.2%), but lower than that of the Actiwatch proprietary IA (6%). Overall, the performance of our proposed deep learning HA is better than other IAs as reported in the literature for wrist actigraphy; a 2019 meta-analysis of data of 49 studies entailing 1582 participants reported the average specificity, sensitivity, and accuracy of sleep scoring to be 51%, 83%, and 82%, respectively, for wrist actigraphy compared to PSG [41].

It is worthy of note that the performance of HA did not significantly differ from PSG in determining SOL (bias of 7.0 min), WASO (bias of −4.2 min), TST (bias of −4.4 min), and SE (bias of −0.7%). We further compared the amount of bias of our proposed deep learning HA against the respective UCSD and Actiwatch proprietary IAs, with respect to the reference PSG. The deep learning HA exhibited significantly lower bias in detecting SOL (respectively, 16.2 min and 43.2 min lower than UCSD and Actiwatch proprietary IAs), WASO (respectively, 28.8 min and 28.2 min lower than UCSD and Actiwatch proprietary IAs), TST (respectively, 12.3-min and 70.1 min lower than UCSD and Actiwatch proprietary IAs), and SE (respectively, 2.5% and 13.6% lower than UCSD and Actiwatch proprietary IAs). Additionally, the amount of MAE for all of the sleep parameters was smallest for the deep learning HA. The amount of bias, in comparison to PSG as reference, in estimating WASO, TST, and SE by the HA was smaller than that reported in the literature and was about the same in estimating SOL; the 2019 meta-analysis of 64 studies that compared the method of actigraphy to that of PSG revealed overestimation of TST by −17.9 min (N = 3437) and SE by −3.8% (N = 2905), and underestimation of SOL by 6.9 min (N = 2534) and WASO by 12.9 min (N = 2537) by the actigraphy method [41]. The amount of bias of the deep learning HA is also smaller than that reported for sleep-staging Fitbit wristband technology, which uses motion, HRV, and respiratory rate to score epochs as sleep vs. wake and estimate sleep parameters [20].

We further assessed the performance of the deep learning HA in detecting REM and NREM sleep stage epochs. The HA showed 40.9% Kappa agreement, 88.1% accuracy, 39.6% sensitivity, and 96.6% specificity with PSG in detecting REM epochs and 54.0% Kappa agreement, 78.9 accuracy, 87.1 sensitivity, and 65.9 specificity in detecting NREM epochs. The total duration of REM sleep was underestimated by the HA by ~30 min and the duration of NREM was overestimated by ~34 min. Since the UCSD and Actiwatch proprietary IAs are only capable of detecting sleep vs. wake epochs, and not sleep stages, it was not possible to compare in this respect the performance of the HA with these two other IAs. Walch et al. [42] tested the performance of their neural network classifier on a MESA dataset and achieved accuracy of ~60–65% in detecting each of the wake, REM, and NREM epochs, which is lower than that found for the HA. Finally, the findings of this study indicate the novel HA, which performs better than existing commercial IAs, can be used as to achieve the sleep/wake scoring of simultaneously sensed heart rate and wrist movement count data of any wearable device.

The strengths of our project in developing the deep learning HA are: (1) use of PSG with only high-quality signals as the reference; (2) reliance on the same actigraphy hardware (Actiwatch), thereby avoiding confounding, to record movement (count) data for the scoring of epochs as sleep or wake by all the three IAs; (3) assessment of a relatively large dataset. The limitations of our project are: (1) use of a PSG/wrist actigraphy database that is representative of older (average age of ~67 years) participants, (2) dependence upon the ECG channel of PSG to derive the HRV metrics, (3) lack of information on the precise start and end times of each of the PSG studies thereby requiring reliance upon SpO2 data as a surrogate indicator of such, and (4) lack of data on the other wrist activity mode measures of ZCM or TAT to incorporate in the proposed deep learning HA to attempt further improvement of its performance.

## 5. Conclusions

The findings of this study show the incorporation of HRV metrics, when the number of epochs is of sufficient number, i.e., duration of the data window is optimal, in combination with movement count data assessed by wrist actigraphy improves the performance of IAs in differentiating the epochs as sleep vs. wake. Application of advanced classifier methods, such as neural network to develop interpretative algorithms, can improve the performance of wrist actigraphy sleep scoring. We proposed a novel deep neural network (Haghayegh Algorithm, HA) that simultaneously incorporates both activity count and HRV metrics to differentiate and score epochs as wake and sleep and improve estimation of parameters of sleep quality. We demonstrated in the study that the HA shows higher accuracy, specificity, and Kappa agreement than the UCSD and Actiwatch proprietary IAs and also in comparison to those values reported in the literature. Estimated sleep onset latency, wake after sleep onset, total sleep time, and sleep efficiency parameters measured by the HA did not differ significantly from the respective reference values produced by PSG; however, it overestimated the total amount of time spent in NREM sleep and underestimated the total amount of time spent in REM sleep.

## Figures and Tables

**Table 1:** Heart rate variability metrics used to develop algorithms [26,27].

Metric	Unit	Definition
***Time Domain***		
Mean R-R	[ms]	Average of R-R intervals per window duration
SDRR	[ms]	Standard deviation of R-R intervals per window duration
Mean HR	[bpm]	Average heart rate per window duration
Min HR	[bpm]	Minimum heart rate calculated using 5 beat moving average per window duration
Max HR	[bpm]	Maximum heart rate calculated using 5 beat moving average per window duration
RMSSD	[ms]	Root mean square of differences between successive intervals per window duration
PNN50	[%]	Number of successive R-R intervals differing by >50 ms divided by total number of R-R intervals per window duration
HRVti	-	Integral of the density of R-R interval histogram divided by height of the histogram per window duration
TINN	[ms]	Baseline width of R-R histogram per window duration
***Frequency Domain***		
VLF Peak	[HZ]	Peak frequency of the very-low-frequency band (0.0–0.04 Hz) per window duration
LF Peak	[HZ]	Peak frequency of the low-frequency band (0.04–0.15 Hz) per window duration
HF Peak	[HZ]	Peak frequency of the high-frequency band (0.15–0.4 Hz) per window duration
VLF Power	[ms^2^]	Absolute power of the very-low-frequency band (0.0–0.04 Hz) per window duration
LF Power	[ms^2^]	Absolute power of the low-frequency band (0.04–0.15 Hz) per window duration
HF Power	[ms^2^]	Absolute power of the high-frequency band (0.15–0.4 Hz) per window duration
LF/HF	-	Ratio between low frequency and high frequency band power per window duration
Stress index	-	Square root of the Baevsky’s stress index per window duration [28]

**Table 2:** Characteristics of the study population (N = 222).

**Age**	67.5 ± 8.3 years
**Gender**	
Male	108 (48.6%)
Female	114 (51.4%)
**Race**	
White, Caucasian	80 (36.0%)
Chinese American	31 (13.9%)
Black, African-American	64 (28.8%)
Hispanic	47 (21.2%)
**Told by doctor as having insomnia**	
Yes	9 (4.1%)
No	213 (95.9%)
**Trouble falling asleep past 4 weeks**	
No, not in the past 4 weeks	127 (57.2%)
Yes, less than once a week	26 (11.7%)
Yes, 1 or 2 times a week	36 (16.2%)
Yes, 3 or 4 times a week	20 (9.0%)
Yes, 5 or more times a week	13 (5.9%)
**Chronotype**	
Definitely a morning type	78 (35.1%)
Rather more a morning than an evening type	60 (27.0%)
Rather more an evening than a morning type	29 (13.1%)
Definitely an evening type	29 (13.1%)
Neither a morning nor an evening type	26 (11.7%)

**Table 3:** Estimated sleep parameters derived by polysomnography (PSG), actiwatch plus its proprietary interpretative algorithm (IA), the UCSD IA, and the proposed deep learning Haghayegh algorithm (HA) across participants of the test dataset.

Sleep Metric	Metric Definition (Units)	Measurement Method	Measured Values Mean (SD)
Sleep Period Time (SPT)	Total recording time (min)	PSG	509.7 (67.1)
Total Sleep Time (TST)	Total time scored as “sleep” (min)	PSG Actiwatch IA UCSD IA Deep Learning HA	367.8 (65.3) 442.3 (62.9) 359.9 (70.2) 372.2 (81.5)
Sleep Efficiency (SE)	(TST/SPT) × 100 (%)	PSG Actiwatch IA UCSD IA Deep Learning HA	72.6 (11.5) 86.9 (6.5) 70.8 (11.0) 73.3 (13.4)
Sleep Onset Latency (SOL)	Duration of time from lights off to first 10 min block when ≥50% of epochs scored as sleep (min)	PSG Actiwatch IA UCSD IA Deep Learning HA	60.6 (39.9) 10.4 (19.5) 37.3 (33.5) 53.6 (42.0)
Wake After Sleep Onset (WASO)	Cumulative amount of time spent in wakefulness after sleep onset (min)	PSG Actiwatch IA UCSD IA Deep Learning HA	82.5 (54.2) 58.5 (32.1) 115.5 (54.4) 86.7 (56.6)
Non-Rapid Eye Movement Sleep (NREM)	Time spent in Sleep Stages N1-N4 (min)	PSG Deep Learning HA	293.7 (55.4) 328.1 (80.7)
Rapid Eye Movement Sleep (REM)	Time spent in Rapid Eye Movement Sleep (min)	PSG Deep Learning HA	74.1 (24.3) 44.2 (25.3)

**Table 4:** Mean and 95% confidence interval of accuracy, sensitivity, specificity, and Cohen’s kappa in scoring the 30-s epochs as sleep vs. wake by the classic deep learning (Residual Network [29,30], Fully Convolutional Neural Networks [29,30], Encoder [29,31], and Time Le-Net [29,32]) Architectures applied to activity count data only and also activity count plus heart rate variability (HRV) data of periods (windows) of different durations.

Features	Accuracy (%)	Sensitivity (%)	Specificity (%)	Cohen’s Kappa (%)
Activity count	69.1 (59.7, 78.5)	68.1 (52.1, 84.1)	71.5 (63.9, 79.1)	39.1 (30.0, 48.2)
Activity count + 30-s HRV	73.3 (65.0, 81.6)	81.6 (66.5, 96.7)	52.5 (40.3, 64.7)	37.4 (28.0, 46.8)
Activity count + 3-min HRV	72.4 (66.0, 78.8)	78.7 (66.1, 91.3)	56.9 (44.8, 69.0)	36.3 (28.8, 43.8)
Activity count + 5-min HRV	74.9 (68.7, 81.1)	79.5 (67.9, 91.1)	63.7 (55.3, 72.1)	43.8 (37.8, 49.8)

**Table 5:** Epoch-by-epoch comparisons of scoring epochs as sleep vs. wake by Actiwatch and its proprietary interpretative algorithm (IA), the UCSD IA, and proposed deep learning Haghayegh algorithm (HA) vs. polysomnography as reference.

Interpretative Algorithm	Accuracy (%)	Sensitivity (%)	Specificity (%)	Cohen’s Kappa (%)
***Sleep detection***				
proprietary	79.2 (77.2, 81.2)	95.5 (94.6, 96.5)	35.7 (32.2, 39.2)	35.7 (31.6, 39.8)
UCSD	77.8 (75.7, 79.8)	83.3 (81.1, 85.5)	61.8 (57.4, 66.1)	42.7 (38.0, 47.4)
Deep learning HA	84.5 (82.6, 86.3)	89.5 (86.9, 92.2)	70.0 (66.4, 73.5)	58.7 (55.0, 62.4)
***REM detection*** ^1^				
Deep learning HA	88.1 (87.0, 89.1)	39.6 (34.8, 44.4)	96.6 (95.9, 97.3)	40.9 (36.1, 45.7)
***Non-REM detection*** ^2^				
Deep learning HA	78.9 (77.4, 80.4)	87.1 (84.1, 90.2)	65.9 (62.7, 69.2)	54.0 (50.8, 57.1)

Values reported as mean and 95% confidence limits in parentheses. ^1^ Detection of rapid eye movement (REM) epochs vs. non-rapid eye movement (NREM) sleep plus wake epochs. ^2^ Detecting NREM epochs vs. REM sleep plus wake epochs.

**Table 6:** Comparison of sleep parameters estimated by Actiwatch proprietary interpretative algorithm (IA), UCSD IA, and deep learning Haghayegh algorithm (HA) vs. polysomnography (PSG) as reference.

Variable (Units)	Overall Bias (95% CI) [*p* Value]	Bland–Altman Limits of Agreement		MAE
		Lower Limit	Upper Limit	
**SOL (min)**				
Actiwatch IA vs. PSG	50.2 (41.0, 59.3) [<0.001]	−29.1	129.5	50.9
UCSD IA vs. PSG	23.3 (13.9, 32.6) [<0.001]	−58.0	104.5	31.8
Deep Learning HA vs. PSG	7.0 (−1.2, 15.2) [0.093]	−63.9	78.0	19.9
*Comparing bias of Deep Learning HA* vs. *bias of Actiwatch IA: d = 43.2, p < 0.001* *Comparing bias of Deep Learning HA* vs. *bias of UCSD IA: d = 16.2, p < 0.001*				
**WASO (min)**				
Actiwatch IA vs. PSG	24.0 (14.6, 33.4) [<0.001]	−57.2	105.2	35.4
UCSD IA vs. PSG	−33.0 (−43.7, −22.4) [<0.001]	−125.2	59.1	44.8
Deep Learning HA vs. PSG	−4.2 (−15.3, 6.9) [0.451]	−100.0	91.5	35.3
*Comparing bias of Deep Learning HA* vs. *bias of Actiwatch IA: d = 28.2, p < 0.001* *Comparing bias of Deep Learning HA* vs. *bias of UCSD IA: d = 28.8, p < 0.001*				
**TST (min)**				
Actiwatch IA vs. PSG	−74.5 (−86.4, −62.7) [<0.001]	−177.1	28.1	76.0
UCSD IA vs. PSG	7.8 (−4.2, 19.8) [0.198]	−95.8	111.5	41.4
Deep Learning HA vs. PSG	−4.4 (−17.0, 8.1) [0.483]	−113.0	104.1	40.1
*Comparing bias of Deep Learning HA* vs. *bias of Actiwatch IA: d = 70.1, p < 0.001* *Comparing bias of Deep Learning HA* vs. *bias of UCSD IA: d = 12.3, p = 0.042*				
**SE (%)**				
Actiwatch IA vs. PSG	−14.3 (−16.5, −12.1) [<0.001]	−33.1	4.5	14.6
UCSD IA vs. PSG	1.8 (−0.6, 4.2) [0.134]	−18.9	22.5	8.2
Deep Learning HA vs. PSG	−0.7 (−3.2, 1.8) [0.584]	−22.1	20.7	7.9
*Comparing bias of Deep Learning HA* vs. *bias of Actiwatch IA: d = 13.6, p < 0.001* *Comparing bias of Deep Learning HA* vs. *bias of UCSD IA: d = 2.5, p = 0.032*				
**REM sleep (min)**				
Deep Learning HA vs. PSG	29.9 (23.0, 36.8) [<0.001]	−29.5	89.3	33.9
**NREM sleep (min)**				
Deep Learning HA vs. PSG	−34.3 (−47.5, −21.2) [<0.001]	−147.7	79.0	55.3

Positive values of bias indicate underestimation by IAs. Abbreviations: d: absolute difference; MAE: Mean Absolute Error; REM: Rapid Eye Movement; NREM: Non-Rapid Eye Movement; SE: Sleep Efficiency; SOL: Sleep Onset Latency; TST: Total Sleep Time; WASO: Wake After Sleep Onset; PSG: Polysomnography.

## Supplementary Materials

The following are available online at https://www.mdpi.com/1424-8220/21/1/25/s1, Table S1: List of layers of the proposed deep learning model. Table S2: Linear regression analysis of trend in Bland-Altman plots, i.e., trend in differences between estimated sleep parameters (y-axis of each depicted Bland–Altman plot) relative to magnitude of the mean per sleep parameter (x-axis of each depicted Bland–Altman plot).

## Code Availability

The deep learning models were developed using freely available libraries in Python. Access to the deep learning HA code and instructions on its use are available from the corresponding author upon request without charge for non-commercial purposes.

## Appendix A

**Table A1:** Equations for calculation of Accuracy, Sensitivity, Specificity, and Cohen’s Kappa.

		Actigraphy Scoring	
		0	1
**PSG Scoring**	0	TN	FP
	1	FN	TP

$$Accuracy=\frac{TP+TN}{TP+TN+FP+FN}$$

$$Sensitivity=\frac{TP}{TP+FN}$$

$$Specificity=\frac{TN}{TN+FP}$$

$$Po=\frac{TP+TN}{TP+TN+FP+FN}$$

$$Pe=\frac{\left(TP+FP\right)*\left(TP+FN\right)+\left(FN+TN\right)*\left(FP+TN\right)}{{\left(TP+TN+FP+FN\right)}^{2}}$$

$$Cohen^{\prime }s\;Kappa=\frac{Po-Pe}{1-Pe}$$
