# Supplementary material for: Deep Neural Network Sleep Scoring Using Combined Motion and Heart Rate Variability Data
Source: Sensors (Basel). 2020 Dec 23;21(1):25. doi: 10.3390/s21010025 (PMC7793092; doi:10.3390/s21010025)
Supplement: Supplementary file 1 [file sensors-21-00025-s001.pdf]

Supplementary Material

Table S1. List of layers of the proposed deep learning model.

Layer type	Output shape
Bach_normalization	(None, 21, 18)
Reshape	(None, 21, 18, 1)
Conv2D	(None, 21, 18, 43)
Bach_normalization	(None, 21, 18, 43)
Activation	(None, 21, 18, 43)
Conv2D	(None, 21, 18, 19)
Bach_normalization	(None, 21, 18, 19)
Activation	(None, 21, 18, 19)
Conv2D	(None, 21, 18, 50)
Bach_normalization	(None, 21, 18, 50)
Activation	(None, 21, 18, 50)
Conv2D	(None, 21, 18, 43)
Bach_normalization	(None, 21, 18, 43)
Activation	(None, 21, 18, 43)
Conv2D	(None, 21, 18, 100)
Bach_normalization	(None, 21, 18, 100)
Activation	(None, 21, 18, 100)
Conv2D	(None, 21, 18, 80)
Bach_normalization	(None, 21, 18, 80)
Activation	(None, 21, 18, 80)
Conv2D	(None, 21, 18, 39)
Bach_normalization	(None, 21, 18, 39)
Activation	(None, 21, 18, 39)
Conv2D	(None, 21, 18, 70)
Bach_normalization	(None, 21, 18, 70)
Activation	(None, 21, 18, 70)
Conv2D	(None, 21, 18, 23)
Bach_normalization	(None, 21, 18, 23)
Activation	(None, 21, 18, 23)
Reshape	(None, 21, 414)
LSTM	(None, 21, 48)
LSTM	(None, 21, 90)
LSTM	(None, 21, 66)
LSTM	(None, 21, 63)
Drop_Out	(None, 21, 63)
Time_Distributed	(None, 21, 4)
Activation	(None, 21, 4)
Lambda	(None, 4)

Table S2: Linear regression analysis of trend in Bland-Altman plots, i.e., trend in differences between estimated sleep parameters (y-axis of each depicted Bland–Altman plot) relative to magnitude of the mean per sleep parameter (x-axis of each depicted Bland–Altman plot)

Variable (Units)	Trend in bias[1]	P value (Significance of trend)
**SOL (min)**		
Actiwatch IA vs. PSG	Bias = 12.86 + 1.05 M	<0.001
UCSD IA vs. PSG	Bias = 10.91 + 0.25 M	0.107
Deep Learning HA vs. PSG	Bias = 10.80 - 0.07 M	0.561
**WASO (min)**		
Actiwatch IA vs. PSG	Bias = -19.21 + 0.61 M	<0.001
UCSD IA vs. PSG	Bias = -32.38 - 0.01 M	0.953
Deep Learning HA vs. PSG	Bias = 0.46 - 0.06 M	0.628
**TST (min)**		
Actiwatch IA vs. PSG	Bias = -92.76 + 0.04 M	0.664
UCSD IA vs. PSG	Bias = 38.8 - 0.09 M	0.385
Deep Learning HA vs. PSG	Bias = 89.23 - 0.25 M	0.006
**SE (%)**		
Actiwatch IA vs. PSG	Bias = -70.53 + 0.71 M	<0.001
UCSD IA vs. PSG	Bias = -2.00 + 0.05 M	0.663
Deep Learning HA vs. PSG	Bias = 12.79 - 0.18 M	0.097
**REM sleep (min)**		
Deep Learning HA vs. PSG	Bias = 33.7 -0.06 M	0.719
**NREM sleep (min)**		
Deep Learning HA vs. PSG	Bias = 101 - 0.44 M	<0.001

[1] Linear regression line of data of Bland-Altman plots; presented as: *Bias= Intercept+slope×mean of measured values (M)*. Abbreviations: NREM: Non-Rapid Eye Movement; REM: Rapid Eye Movement; SE: Sleep Efficiency; SOL: Sleep Onset Latency; TST: Total Sleep Time; WASO: Wake After Sleep Onset; PSG: Polysomnography
